# Bibliographical Department

**Published:** 1881-09

**Authors:** 


### BIBLIOGRAPHICAL DEPARTMENT.

"Nothing extenuate nor set down aught in malice."

*Presented to the Delegates to the International Medical Congress, London*, 1881, *with the Compliments of William Wood & Company, Medical Publishers, New York.* We are indebted to the Messrs. Wood for a copy of this most beautifully gotten up Directory of the proceedings of the International Medical Congress. First, we have the daily programme of the congress, including the general arrangement of sections, informal dinner at the Crystal Palace, excursions, etc., etc. Next, historical sketch of the house of William Wood & Company, and catalogue of their large and valuable medical publications. Then follows the prospectus of the great surgical work of the age, in course of publication by the Messrs. Wood, to be known as the "Encyclopædia of Surgery." A systematic treatise of the Theory and Practice of Surgery, by authors of various nations, edited by John Ashurst, Jr., M. D., Professor of Clinical Surgery in the University of Pennsylvania, in six volumes, royal octavo, illustrated with chromo-lithographs and wood engravings.

Then the subjects and contents of the several volumes follow, and the names of the principal contributors at home and abroad appear.

We frankly confess we like the plan of the work, and accept the general arrangement of subjects ; and believe the editorial department will receive all the attention its vast importance requires at the hands of Prof. Ashurst ; and we think it would be difficult indeed, if not impossible, to have gotten better and more thoroughly qualified contributors for its pages than the majority of the names that are mentioned as such. Yet we must confess that we feel sure that several equally as good, if not better surgeons, both theoretical and practical, could have been found — as they certainly exist — in certain portions of the United States, as are a few whose names appear among the contributors.

But, of course, it would have added to the size of the work to increase the list of contributors to a large extent. As it is, the work will probably be the fullest in the language, when completed. It is far better, for various reasons, that such a treatise on surgery should be the work of several able and experienced practitioners, than of a single man, however eminent and well fitted to practice surgery he might be. We cordially extend our best wishes for the success of the enterprise, and will cheerfully bring it to the notice of our readers from time to time as occasion may occur for doing so.

*The Microscope and its Application to Medicine and Pharmacy.* Edited and published by Charles H. Stowell, M. D., Assistant Professor of Physiology and Histology, Louisa Reed Stowell, W. S.,Assistant in Microscopical Botany, University of Michigan. An illustrated bi-monthly journal, Ann Harbor, Mich. Terms $1.50 U. S., and Great Britain 5 shillings.

Notwithstanding the great redundancy of the periodical publications relating to Medicine and the collateral sciences, we think there is room for *The Microscope*, and we feel that sure it will be, as it certainly ought to be, well sustained as soon as its merits are known and appreciated. So far as we know, the field it has chosen for its labors is occupied by it alone.

*Fort Wayne Journal of the Medical Sciences. Edited by Professors W. H. Gobretch and G. W. McCaskey*; $2 per annum; Fort Wayne, Ind. Vol. 1, No. 1, has been received, and is well filled with interesting professional reading matter. We wish it success.

*Report of the Trial of James Thomas De Jannetta, for Homicide, with an Appendix. By Eugene Grisson, M. D., L. L. D., Raleigh, N. C.* We have read the testimony in this unfortunate case, and feel assured that it ably and clearly and justly released the prisoner from the strong arm of the law.

*Abortive Treatment of Pneumonia. By W. Y. Godbury, M. D., of Yazoo City, Miss.* Quite an interesting pamphlet.

*Cartwright Prize Essay. Observations with the Haemaegtometer upon the globular composition of the blood and milk. By Frederick P*

*Henry, M. D., physician to the Hospital of the Protestant Episcopal Church, Philadelphia, etc., etc. E. Scintilla, Lux.* We acknowledge our indebtedness to the author for a copy of this most instructive and interesting essay.

*An Open Letter to the National Board of Health, By Robt. B. S. Hargis, M. D., on Yellow Fever,* Has been received. The Doctor defends his views of the origin of the disease with considerable force.

*The Baltimore College of Dental Surgery* sends us its forty-second annual catalogue. This time-honored institution (the first established in the world) conferred the degree of D.D.S. on fifty-three students, which is the largest graduating class since the founding of the college.

This summer it has moved into new and more commodious quarters which are elegant and admirably adapted for its purposes. 'Tis said to be "beyond doubt, the most complete and handsome building devoted to dental education in the world."

*The Pennsylvania College of Dental Surgery* compliments us with their twenty-sixth annual announcement. This catalogue shows an able corps of trustees, faculty, clinical instructors and sixty-four graduates—session 1880–81.

*Meetings of Dental Societies in September.*—The Nebraska State Dental Society on the 12th at Omaha; The Maryland and District of Columbia Dental Society on the 7th in the Baltimore College of Dental Surgery, at Baltimore.

